# Uric Acid as a Potential Peripheral Biomarker for Disease Features in Huntington’s Patients

**DOI:** 10.3389/fnins.2020.00073

**Published:** 2020-03-04

**Authors:** Jody Corey-Bloom, Ameera Haque, Sameer Aboufadel, Chase Snell, Ryan S. Fischer, Steven W. Granger, Douglas A. Granger, Elizabeth A. Thomas

**Affiliations:** ^1^Department of Neurosciences, University of California, San Diego, La Jolla, CA, United States; ^2^Salimetrics, LLC, Carlsbad, CA, United States; ^3^Institute for Interdisciplinary Salivary Bioscience Research, University of California, Irvine, Irvine, CA, United States; ^4^School of Medicine, Johns Hopkins University, Baltimore, MD, United States; ^5^Bloomberg School of Public Health, Johns Hopkins University, Baltimore, MD, United States; ^6^School of Nursing, Johns Hopkins University, Baltimore, MD, United States; ^7^Department of Neuroscience, The Scripps Research Institute, La Jolla, CA, United States

**Keywords:** neurodegenerative, biomarker, peripheral, brain, blood, saliva, biofluid

## Abstract

Oxidative stress has long been implicated in the pathophysiology and progression of Huntington’s disease (HD). Uric acid (UA) is a naturally occurring antioxidant that is present in the brain and periphery. Growing evidence has implicated UA as a molecular biomarker for several neurodegenerative diseases, most notably Parkinson’s disease (PD). In this study, we investigated UA levels in clinical samples from HD patients and normal controls (NCs) and assessed potential relationships between UA levels and disease and clinical data. UA levels were measured in plasma (*n* = 107) and saliva (*n* = 178) samples from premanifest (pre-HD) and manifest HD patients and control subjects. Gender effects of UA levels were observed in both biofluids, with male patients showing higher UA levels compared to female patients. Comparisons of UA levels across diagnostic groups, separated by gender, revealed that both plasma and salivary UA levels were significantly lower in female pre-HD and manifest HD patients compared to NCs. Salivary levels of UA were also significantly lower in male manifest HD patients versus controls, but not in plasma. Correlations of peripheral UA levels to clinical data also showed differences according to gender. In male HD patients, both plasma and salivary UA levels were significantly negatively correlated with total functional capacity (TFC), while positive correlations were observed with total motor score (TMS). Female HD patients showed a significant positive correlation between plasma UA levels and TMS, while salivary UA levels from female patients were significantly correlated to disease burden. Finally, in a separate cohort, we show that UA levels are decreased in postmortem prefrontal cortical samples (*n* = 20) from HD subjects compared to matched controls. These findings suggest that decreased levels of UA in the brains of HD patients can be reflected in peripheral fluids, with salivary measures of UA particularly offering significant promise as a potentially relevant, non-invasive biomarker of disease symptoms and burden. Our findings further highlight the impact of sexual dimorphism in HD pathophysiology.

## Introduction

Huntington’s disease (HD) is an inherited, progressive neurodegenerative disorder caused by a CAG repeat expansion in the 5′ coding region of the Huntington (*HTT*) gene (Huntington Disease Collaborative Research Group, 1993). Despite enormous progress in our understanding of this disease, the mechanisms connecting mutant huntingtin (Htt) protein with cell death and pathological symptoms remain unclear. Oxidative damage, mitochondrial dysfunction, and impairment in the electron transport chain have been suggested to have important roles in the degenerative process in HD (Browne et al., 1997; Trushina and McMurray, 2007; Franco-Iborra et al., 2018; Zheng et al., 2018). Specifically, past studies have demonstrated enhanced oxidative stress and oxidative DNA damage in peripheral blood from HD patients (Chen et al., 2007), in cultured fibroblasts from HD patients, in HD mouse models (Goula et al., 2009), and in striatal cells derived from HD knock-in mice (Li et al., 2010; Ribeiro et al., 2012). Oxidative damage is not considered to be substantial in the early stages of HD, but rather is proposed to be a major contributor to pathology as the disease progresses (Johri and Beal, 2012). HD symptoms typically appear between the ages of 35 and 55, although juvenile forms of the disease exist, which present before the age of 20 (van Dijk et al., 1986), and the disease progresses over 10–20 years from a presymptomatic state to complete disability and death. The age of onset for HD varies inversely with the length of the disease-causing CAG repeat mutation, with the threshold being between 36 and 39 triplet repeats (Andresen et al., 2007). However, the age of onset and its progression vary considerable among patients, even among patients with identical CAG repeat lengths (Andrew et al., 1993; Wexler et al., 2004; Andresen et al., 2007), warranting the need for biomarkers that might help predict onset. In addition, gender has emerged as another factor that can affect disease outcomes in HD, with women showing worse symptoms and a faster rate of progression (Dorner et al., 2007; Zielonka et al., 2013). In fact, there are several gender-related differences that have been reported in other neurodegenerative diseases as well, which have enhanced our current understanding of the impact of sexual dimorphism in neurological diseases and the implications for preventive and therapeutic outcomes (Zagni et al., 2016; Ullah et al., 2019).

Uric acid (UA), an endogenous compound that is formed as the end-product of purine metabolism, is a powerful antioxidant in the CNS and periphery. In fact, UA is the most abundant natural antioxidant in human blood. Although imbalanced UA levels have a long-standing association with medical conditions such as gout, UA also affects the central nervous system and has been specifically been linked to neurological conditions (Bowman et al., 2010; Fang et al., 2013). With respect to neurodegenerative diseases, the interest in UA was initially sparked by evidence of oxidative damage in another movement disorder, Parkinson’s disease (PD). Previous work in PD has demonstrated low levels of UA in postmortem substantia nigra (Church and Ward, 1994; McFarland et al., 2013), as well as in blood from PD patients (Davis et al., 1996; Schlesinger and Schlesinger, 2008). Since these early observations, accumulating epidemiological studies, laboratory data, preclinical models, and early clinical trial results have provided substantial support for a neuroprotective role for UA and its potential as a disease biomarker for PD (Constantinescu and Zetterberg, 2011; Chen et al., 2012; Crotty et al., 2017). In particular, UA has been suggested as a promising biomarker of reduced risk and milder progression in PD (Schlesinger and Schlesinger, 2008; Paganoni and Schwarzschild, 2017; Wen et al., 2017).

Because oxidative stress represents a shared pathological mechanism, not only between PD and HD but also across several neurodegenerative diseases, we hypothesized that UA could also serve as a potentially relevant biomarker in HD. Hence, in this study, we measured UA levels in peripheral fluids and postmortem brain samples from HD patients and assessed their potential relationships with disease and clinical data. We include saliva in this study as a non-invasive alternative to blood, and a biofluid that has the potential to expand biomarker measurements in diverse settings.

## Materials and Methods

### Human Subjects

This study was approved by the University of California, San Diego (UCSD) Institutional Review Board in accordance with the requirements of the Code of Federal Regulations on the Protection of Human Subjects. Informed consent from all subjects was obtained before their participation in accordance with the Declaration of Helsinki. Patients were recruited from the UCSD HDSA Center of Excellence. HD patient criteria included a definitive diagnosis of HD with family history and an expanded trinucleotide CAG repeat of 40 or more. Normal controls (NCs) had no reported history of neurological conditions, psychiatric disorders, or gout, and no use of psychoactive substances or medications. Demographic information was collected at the time of saliva collection, including sex, age, CAG repeat length, and age of onset. This information is provided in Table 1. Patients were assessed for cognitive and motor function using the Mini-Mental State Examination (MMSE; score range, 0–30) (Gluhm et al., 2013), the Unified Huntington’s Disease Rating Scale (UHDRS) total motor score (TMS; range, 0–124), and total functional capacity (TFC; range, 0–13). Overall genetic or disease burden was assessed using the disease burden score (DBS). These data are summarized in Supplementary Table S1.

**TABLE 1:** Summary of subjects used for these studies.

	HD	Pre-HD	NC	Total no.
**Plasma**				
Number of patients	38	31	38	107
Female/male	22:16	18:13	18:20	58:49
Average age (years)	59.5 ± 13.1	43.7 ± 13.8	59.9 ± 14.3	
Average CAG repeat	42.7 ± 2.8	41.3 ± 1.9	NA	
**Saliva**				
Number of patients	45	49	84	178
Female/male	29:16	26:23	41:43	96:82
Average age (years)	56.5 ± 13.4	44.8 ± 12.2	52.9 ± 16.02	
Average CAG repeat	43.5 ± 2.8	41.0 ± 1.7	NA	
**Postmortem brain**				
Number of subjects	10	10		20
Female/male	3:7	3:7		6:14
Average age (years)	59.6 ± 4.33	60.0 ± 4.32		
Average PMI	13.5 ± 5.08	16.8 ± 9.1		

*PMI, postmortem interval; HD, Huntington’s disease; Pre-HD, premanifest; NC, normal controls.*

### Plasma Collection

Blood from consenting HD patients and controls was drawn by venipuncture into 2 ml lavender/ethylenediaminetetraacetic acid (EDTA) tubes. EDTA/whole blood was mixed well by inversion and spun at 900 × *g* for 15 min. The top plasma layer was transferred into 4 × 1 ml aliquots and snap frozen and stored at −80°C. All subjects who provided a plasma sample also provided a saliva sample.

### Saliva Collection

All donors were asked to refrain from smoking, eating, drinking, or oral hygiene procedures for at least 1 h before sample collection and then rinsed their mouth thoroughly with water 15–20 min before sample collection. Saliva samples were collected between 10:00 AM and 4 PM using the passive drool method according to previously established protocols (Granger et al., 2012). Roughly 2–3 ml of unstimulated whole saliva was obtained. Besides the patients who provided a plasma sample, an additional group of patients provided a saliva-only sample. Samples were immediately frozen at −20°C at the time of collection and then stored at −80°C. At the time of use, saliva samples were thawed and centrifuged at 10,000 × *g* for 10 min at 4°C to remove insoluble material and cellular debris. Supernatants were collected and used for all assays.

### Postmortem Brain Samples

Prefrontal cortical tissue was obtained postmortem from subjects with HD cases and age-matched NCs with no history of psychiatric illness. Tissues were obtained from the National Institutes of Health (NIH) NeuroBioBank^1^. All HD cases showed a moderately to severely atrophied cortical tissue with pathological grades 2–4. Exact CAG repeat numbers were not available. Details such as Vonsattel grading, age, gender, and postmortem delay can be found in Table 1. Frozen tissue samples (∼1 g) were homogenized in lysis buffer and then centrifuged at 10,000 × *g* at 4°C for 20 min. Supernatant fractions were used for UA assays below. Total protein levels in each sample were quantified by the bicinchoninic acid (BCA) protein assay (Abcam). Brain samples were obtained from a separate cohort of subjects than those providing plasma and/or saliva samples above.

### UA Measurements

Uric acid levels in all samples were measured using a commercially available colorimetric enzymatic reaction kit (Catalog No. 1-3802, Salimetrics, CA, United States). The amount of UA in the sample was assessed by measuring the production of red chromogen during an enzymatic reaction process. All assays were performed in duplicate by operators blinded to the clinical state of the participant. The assay used 10 μl of sample per well and had a range of sensitivity from 0.78 to 25 mg/dl.

### Statistics

The distribution of the data values in brain samples and each peripheral fluid was tested for normality using the Kolmogorov–Smirnov normality test to determine whether parametric or non-parametric tests would be used for correlation variables. Outlier values were determined using either the Grubb’s test for sex and age comparisons or the Iglewicz and Hoaglin’s robust test for multiple outliers (two-sided test; *z* = 3.5) for diagnostic comparisons, resulting in the removal of two datapoints for the plasma samples and four datapoints for the saliva samples. A one-way analysis of covariance (ANCOVA) was used to determine if differences in diagnostic groups were associated with age or gender. Group differences separated by sex were determined by one-way ANOVA, followed by Bonferroni or Tukey’s multiple comparison test. Linear regression analysis (Pearson or Spearman correlation) was used to compare UA levels against disease and clinical variables. Differences between sex and postmortem interval (brain samples only) were determined using Student’s *t*-test (unpaired; two-tailed). Statistical analyses were performed using JASP STATs and Prism.

## Results

### Plasma UA in HD Patients

**FIGURE 1:**
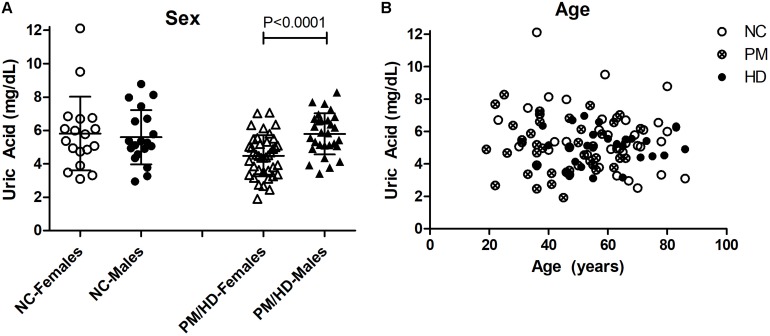
Gender and age effects of uric acid (UA) in plasma from Huntington’s disease (HD) patients and normal controls (NC). **(A)** Significant differences in gender were determined by Student’s *t*-test, unpaired; two-tailed; *p* < 0.0001. The HD patients include pre-HD (PM) and manifest HD patients. **(B)** Plasma UA levels were not significantly associated with age in any group.

We measured UA concentrations in plasma from patients with manifest HD (*n* = 38), premanifest HD (designated “pre-HD”) (*n* = 31), and healthy NC individuals with no history of neurological or psychiatric disorders (*n* = 38) (Table 1). There was no significant difference in plasma UA levels between genders in control subjects, although levels were significantly higher in male compared to female patients in pre-HD and HD subjects (Figure 1A) consistent with previous studies (Andreadou et al., 2009; Kivity et al., 2013; Riis et al., 2018; Yang et al., 2019). Gender ratios across the three groups of subjects were not significantly different. The pre-HD group was statistically significantly younger than both the controls and manifest HD groups (ANOVA *p* < 0.001); however, when both age and gender were considered as covariates in determining the difference between diagnostic groups using ANCOVA, the age effect was not significant (*p* < 0.193), with the gender effect remaining (*p* < 0.006). Accordingly, linear correlation revealed no significant effects of age on UA levels (Figure 1B).

**FIGURE 2:**
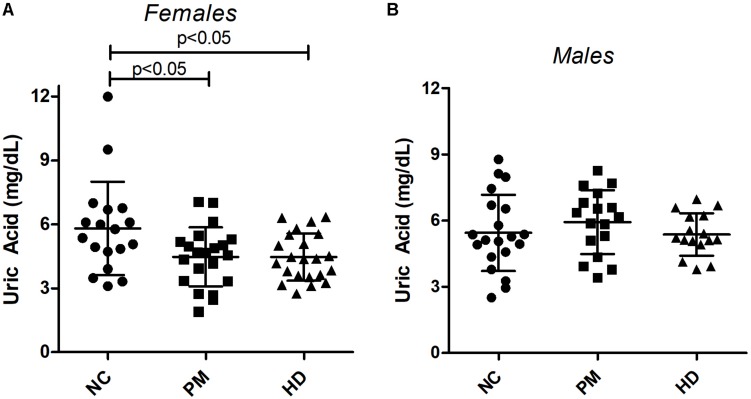
Changes in plasma uric acid (UA) levels by gender and diagnostic group. UA levels were measured in premanifest [pre-Huntington’s disease (PM), manifest HD and normal control (NC) subjects in females **(A)** and males **(B)**. Significant differences among diagnostic groups were determined using one-way ANOVA, followed by Bonferroni posttest to compare all groups; *p* < 0.05. Bars reflect the mean uric acid levels ± SD.

Owing to the significant gender effect, we compared UA levels across diagnostic groups in male and female HD patients separately. Using ANOVA, we found that female subjects showed significantly lower levels of UA in both pre-HD and manifest HD patients versus NCs (ANOVA *p* < 0.014; *p* < 0.05 for NC vs. pre-HD and NC vs. HD; Figure 2A). Similar decreases were not observed in male patients (ANOVA *p* < 0.469; Figure 2B).

We next correlated UA levels with clinical data, keeping the gender distinction. In male manifest HD patients, plasma UA levels were significantly negatively correlated with TFC (Pearson *r* = −0.600; *p* = 0.014) and positively correlated with TMS from the UHDRS (Pearson *r* = 0.644; *p* = 0.007) (Table 2). In female manifest HD patients, plasma UA levels were positively correlated with TMS (Pearson *r* = 0.656; *p* = 0.005), similar to the effect observed in male patients (Table 2). No significant correlations were detected between plasma UA levels and clinical or disease metrics in pre-HD patients of either gender.

**TABLE 2:** Correlations between plasma uric acid and clinical and disease data in female and male HD patients.

	Female				Male			
	Premanifest HD		Manifest HD		Premanifest HD		Manifest HD	
	Pearson/Spearman rho value	*p*-Value	Pearson/Spearman rho value	*p*-Value	Pearson/Spearman rho value	*p*-Value	Pearson/Spearman rho value	*p*-Value
DBS	0.439	0.077	–0.012	0.944	0.306	0.310	0.171	0.526
TFC	0.274	0.071	–0.133	0.426	–0.207	0.498	**−0.600**	**0.014**
MMSE	0.231	0.959	–0.093	0.579	–0.152	0.620	–0.179	0.508
TMS	0.075	0.690	**0.656**	**0.005**	0.029	0.926	**0.644**	**0.007**
CAG repeat no.	0.533	0.057	–0.087	0.621	0.380	0.353	–0.064	0.820
Age of Onset	NA	NA	0.296	0.180	NA	NA	0.171	0.527
Disease duration	NA	NA	0.140	0.402	NA	NA	0.100	0.714

*Values in bold denote statistically significant correlations. DBS, disease burden score; TFC, total functional capacity score; MMSE, Mini-Mental State Exam; TMS, total motor symptom score.*

### Measures of UA in Saliva From HD Patients

**FIGURE 3:**
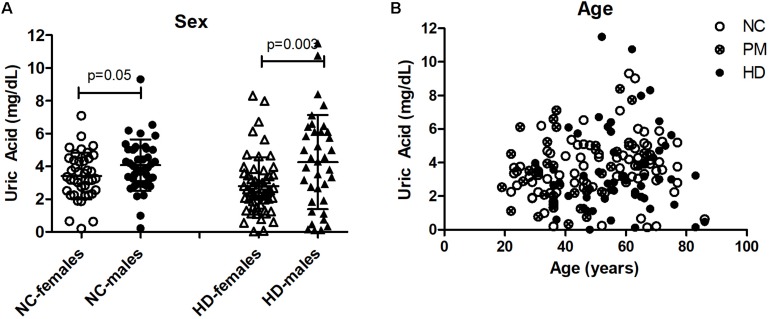
Gender and age effects of uric acid (UA) in saliva from Huntington’s disease (HD) patients and normal controls (NC). **(A)** UA levels were significantly higher in males compared to females in both NC and HD groups. The HD group also included pre-HD patients (PM). Significant differences were determined by Student’s *t*-test, unpaired; two-tailed; *p* = 0.05; *p* = 0.003. **(B)** No significant effects of age on salivary UA levels were observed in any group.

We next assessed UA levels in saliva from pre-HD patients (*N* = 49), HD patients (*n* = 45), and control subjects (*n* = 84) (Table 1). We found that salivary UA levels were significantly higher in male compared to female patients in both cohorts (Figure 3A), consistent with previous studies on saliva (Riis et al., 2018). Similar to the plasma cohort, the pre-HD patients were statistically significantly younger than both the controls and manifest HD groups (ANOVA *p* < 0.0001); however, age was not considered a significant covariate in determining the difference between diagnostic groups using ANCOVA (*p* < 0.350). Accordingly, linear correlation revealed no significant effects of age on salivary UA levels in any diagnostic group (Figure 3B).

**FIGURE 4:**
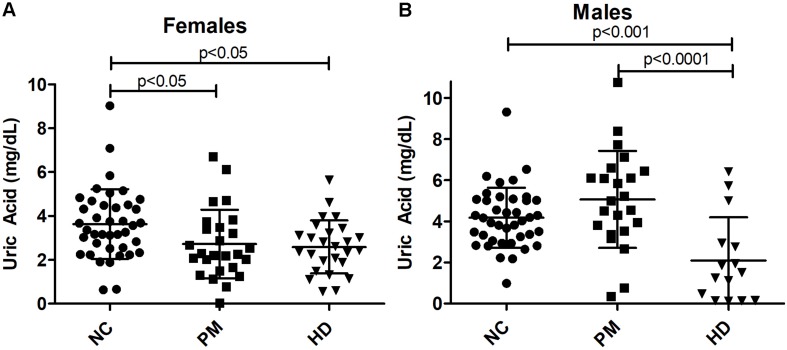
Alterations in salivary uric acid (UA) levels according to diagnostic group in females **(A)** and males **(B)**. Uric acid levels are shown according to diagnostic group, premanifest [pre-Huntington’s disease (PM), manifest HD (HD), and normal controls (NC). Significant differences among diagnostic groups were determined using one-way ANOVA, followed by Bonferroni posttest to compare all groups: *p* < 0.05, *p* < 0.001, *p* < 0.0001, as shown. Bars reflect the mean uric acid levels ± SD.

Owing to the significant gender effect, we compared salivary UA levels across diagnostic groups in male and female HD patients separately. We found that salivary UA levels were significantly lower in female pre-HD and HD patients compared to NCs, with nearly a 40% reduction in UA concentration detected in HD patients (ANOVA *p* < 0.0048; *p* < 0.05 for NC vs. pre-HD and NC vs. HD, Figure 4A). In male patients, UA levels were significantly lower in manifest HD patients compared to controls and pre-HD subjects (ANOVA *p* < 0.0001; *p* < 0.001 for NC vs. pre-HD and NC vs. HD, respectively, Figure 4B).

Comparing to clinical data, we found that salivary UA levels in male HD patients showed the same effects as plasma UA levels, being significantly negatively correlated with TFC (Pearson *r* = −0.617; *p* = 0.012) and positively correlated with TMS (Pearson *r* = 0.627; *p* = 0.009) (Table 3). In female HD patients, salivary UA levels were significantly correlated with DBS (Pearson *r* = −0.434; *p* = 0.009), CAG repeat length (Spearman *r* = −0.397; *p* = 0.018). No significant correlations were observed between salivary UA levels and any clinical or disease metric in pre-HD patients of either gender, consistent with the findings from blood.

**TABLE 3:** Correlations between salivary uric acid and clinical and disease data in female and male HD patients.

	Females				Males			
	Premanifest HD		Manifest HD		Premanifest HD		Manifest HD	
	Pearson/Spearman rho value	*p*-Value	Pearson/Spearman rho value	*p*-Value	Pearson/Spearman rho value	*p*-Value	Pearson/Spearman rho value	*p*-Value
DBS	0.257	0.186	**−0.435**	**0.009**	–0.020	0.943	0.444	0.129
TFC	0.187	0.330	0.035	0.844	0.173	0.521	**−0.617**	**0.012**
MMSE	–0.234	0.221	0.209	0.236	–0.412	0.113	–0.339	0.257
TMS	0.157	0.418	–0.090	0.605	0.019	0.945	**0.627**	**0.009**
CAG repeat #	0.052	0.848	**−0.397**	**0.018**	–0.004	0.990	0.538	0.058
Age of Onset	NA	NA	0.186	0.285	NA	NA	–0.379	0.202
Disease duration	NA	NA	0.104	0.553	NA	NA	–0.111	0.718

*DBS, disease burden score; TFC, total functional capacity score. MMSE, mini-mental state exam; TMS, total motor symptom score. Values shown in bold denote statistically significant correlations.*

### Plasma UA Levels Versus Salivary UA Levels

**FIGURE 5:**
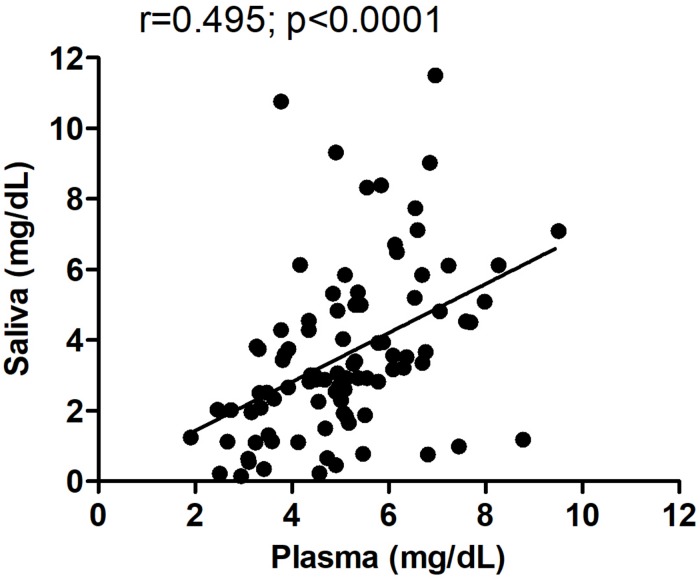
Correlation between plasma and salivary levels of uric acid (UA). The correlation between plasma and salivary levels of UA was determined using Spearman correlation analysis. Pearson *r* = 0.495; *p* < 0.0001. The same subjects provided paired saliva and plasma samples; however, for some subjects, the saliva and plasma were collected on different days spaced up to a week apart.

All subjects who donated a plasma sample also provided a saliva sample; hence, we could directly compare levels of UA in both peripheral fluids in the same individuals. In both male and female normal subjects, plasma levels of UA were higher than those found in saliva (Student’s *t*-test; *p* < 0.0001), consistent with previous studies (Riis et al., 2018). We next examined whether levels of UA in blood correlated with those detected in saliva in all pre-HD and HD patients and found a significant correlation between UA levels in plasma versus saliva (Spearman *r* = 0.495; *p* < 0.0001) (Figure 5), suggesting that salivary UA levels originate, at least in part, from blood sources.

### Uric Acid Levels in HD Postmortem Brain

**FIGURE 6:**
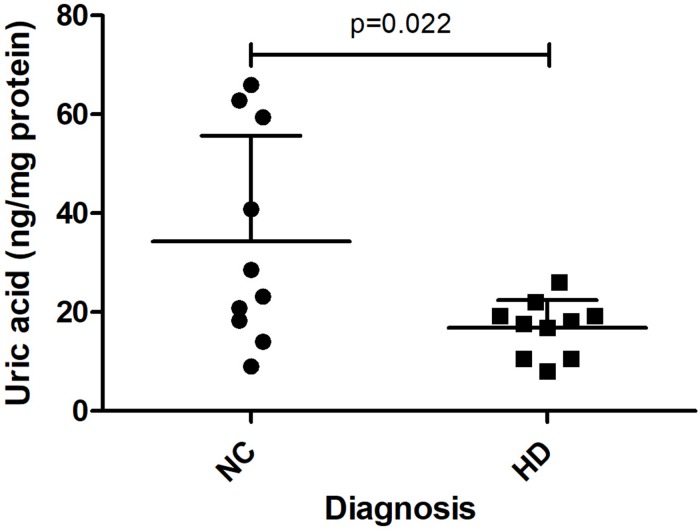
Uric acid (UA) levels in postmortem prefrontal cortex from Huntington’s disease (HD) subjects and matched controls. UA levels, corrected for total protein, were measured in prefrontal cortical samples from *n* = 10 HD patients and *n* = 10 matched normal controls (NC). Data points reflect mean + SD. Difference in UA concentrations in HD versus normal controls was determined using Student’s *t*-test (unpaired, two-tailed), *p* = 0.022.

In a separate study, we measured UA levels in prefrontal cortex obtained postmortem from 10 HD patients and 10 healthy age-matched NCs. The demographic data for these subjects are shown in Table 1. There were no significant differences in mean age, postmortem interval, or sex ratios between the HD and control groups. UA measurements were normalized by total protein levels in each sample to account for differences in starting amounts of tissue. Regression analyses revealed no significant correlations between UA and age or postmortem interval in either group (Supplementary Table S2), although a trend toward a significant correlation with age was observed in the control group (Supplementary Table S2). Comparing between diagnostic groups revealed a significant decrease in UA levels in prefrontal cortex from HD patients compared to NCs (16.8 ± 5.6 vs. 34.3 ± 21.4 ng/mg total protein) (Figure 6). This difference was not affected by sex or age as determined by ANCOVA (*p* = 0.270 and *p* = 0.337 for sex and age, respectively).

## Discussion

Despite accumulating evidence for enhanced oxidative stress and oxidative damage in HD (Chen et al., 2007; Goula et al., 2009; Li et al., 2010; Ribeiro et al., 2012), the major pathways and the sequence of oxidative stress-associated events in HD pathophysiology are poorly understood. Nonetheless, as an endogenous antioxidant, UA represents a potentially relevant biomarker for studies in HD. In this study, we show that UA levels are significantly lower in the plasma and saliva of HD patients compared to healthy controls. This decrease in UA was also detected in postmortem brain samples from HD patients, which is consistent with a previous study that found lower UA levels in postmortem frontal lobe and striatum from HD subjects using high-resolution mass spectrometry (Graham et al., 2016). However, our findings further demonstrate that decreased UA concentrations are also detected in peripheral fluids, including the non-invasive biofluid, saliva. This could possibly reflect a reduced antioxidant capacity in HD that is manifested throughout the body.

Importantly, our results highlight important gender differences in UA levels and the utility for UA to serve as a potential disease biomarker. There are a number of gender-related differences that have been reported in neurodegenerative diseases (Ullah et al., 2019), which have made it necessary to incorporate sex as a biological factor in experimental and clinical studies. Importantly, such studies have enhanced the current understanding of the impact of sexual dimorphism in neurological diseases (Zagni et al., 2016; Ullah et al., 2019). In our studies, we first found that UA levels were higher in male compared to female patients in all of the biospecimens we tested. This is consistent with several past studies, where it has been shown that men have higher levels of UA than women in blood and saliva (Andreadou et al., 2009; Kivity et al., 2013; Riis et al., 2018; Yang et al., 2019). The reason for this difference is not clear, but possible explanations include alcohol consumption, which is higher in men, and the influence of estrogens to lower UA (Jung et al., 2018; Yang et al., 2019). Next, we found gender differences in the ability of UA to distinguish diagnostic groups. In female patients, plasma and salivary levels of UA were significantly lower in both pre-HD and manifest HD patients, while in male patients, UA concentrations were only lower in the saliva of manifest HD patients. The higher levels found in the saliva from male patients, in general, might mask a potential disease effect of UA tested here.

Or there may be a true difference in the role of UA in HD pathology in male vs. female patients. For example, a study examining the relationship between gender and progression of the disease showed that gender played a significant role in the neuropathological changes and phenotypical expression in HD (Dorner et al., 2007). Another study also found a gender influence in disease severity with women displaying poorer motor and functional UHDRS scores (Zielonka et al., 2013). Hence, it is possible that disease-propagating mechanisms are different in men and women, and our studies would suggest an involvement of oxidative stress in these mechanisms. We found that in male HD patients, both plasma and salivary UA levels were significantly positively correlated with TMS and negatively correlated with TFC. The direction of these correlations might appear at odds with the decreased levels of UA detected in HD patients compared to NCs, which would suggest that a reduced antioxidant capacity is detrimental. However, within the HD population, our results would suggest that higher UA levels are more damaging. It is known that too much UA can have negative consequences, such as proinflammatory effects, associated with increased cytokines (Fang et al., 2013). This is consistent with the literature demonstrating increase proinflammatory markers, including interleukin 6 and 1B, in the plasma of premanifest HD patients and in presymptomatic HD mice (Dalrymple et al., 2007; Chang et al., 2015; Politis et al., 2015).

Our findings in plasma are consistent with past literature in other neurodegenerative disorders, where lower blood and brain levels of UA have been reported in PD, ALS, and Alzheimer’s disease patients (Church and Ward, 1994; Constantinescu and Zetterberg, 2011; Chen et al., 2012; Crotty et al., 2017). In PD, correlations were observed between higher serum levels of UA and not only a lower risk of developing the disease but also a slower rate of disease progression (Schlesinger and Schlesinger, 2008; Paganoni and Schwarzschild, 2017; Wen et al., 2017). Interestingly, studies in PD have also reported differences in men and women, with one study showing correlations between serum UA levels and disease duration in patients with PD only in men (Andreadou et al., 2009). Other studies have implicated serum UA as a biomarker for coronary artery disease only in women (Sun et al., 2019).

Our findings in saliva are novel. Biomarker research in saliva has grown over the past few years (Corey-Bloom et al., 2018), largely due to its non-invasive nature and ease of collection in any setting. Here, we have shown that the UA data in saliva mimic several features of plasma and that levels of salivary UA are correlated with those found in plasma. This would suggest that at least some of the UA present in saliva originates from blood sources. However, salivary levels of UA could also arise from leukocytes and buccal cells present in saliva, or the salivary glands themselves, which are innervated by the glossopharyngeal and facial nerves, suggesting potential neural release of UA in saliva.

Potential confounds of our study could be the effects of diet and stress, which could differ between patients and controls. It is known that UA levels can vary according to diet (Zgaga et al., 2012; Towiwat and Li, 2015), and stress and strenuous exercise have been shown to elevate UA (Gonzalez et al., 2008). However, previous studies have demonstrated that, while there is some “state specific” variation in salivary UA levels, the majority of the variance in salivary UA can be attributable to a “trait-like” stable component of the variance (Riis et al., 2018). In addition, the potential effects of medications should be considered in future studies on saliva. For example, some medications can affect UA levels (Caspi et al., 2000) or can cause xerostomia, reducing salivary output.

The utility of salivary measures to reveal information about HD disease features is especially exciting, given the non-invasive nature of saliva collection, although this is a new field with respect to neurological disorders. UA could be an important potential biomarker of disease symptoms and genetic burden, albeit in a gender-specific manner. The fact that the decrease in UA levels in the CNS can be revealed in peripheral fluids is especially exciting and should encourage future studies investigating UA levels in cerebrospinal fluid (CSF) from HD patients.

## Data Availability Statement

All clinical data generated for this study are included in the article/Supplementary Material.

## Ethics Statement

The studies involving human participants were reviewed and approved by the University of California, San Diego Institutional Review Board in accordance with the requirements of the Code of Federal Regulations on the Protection of Human Subjects. The patients/participants provided their written informed consent to participate in this study.

## Author Contributions

JC-B designed the research plan, recruited all subjects for this study, and collected demographic and clinical data from HD patients. AH collected and organized clinical data, performed statistical analyses, and collected saliva samples from HD patients. SA collected clinical data from HD patients. CS collected saliva samples from HD patients. RF performed the experiments. SG provided reagents. DG provided reagents. ET designed the research plan, performed experiments, performed statistical analyses, and wrote the manuscript.

## Conflict of Interest

DG is the founder and Chief Scientific and Strategy Advisor at Salimetrics LLC and Salivabio LLC. The nature of these relationships is managed by the policies of the committees on conflict of interest at Johns Hopkins University School of Medicine and the University of California at Irvine. RF and SG were also employed by Salimetrics LLC. The remaining authors declare that the research was conducted in the absence of any commercial or financial relationships that could be construed as a potential conflict of interest.

## Footnotes

1 https://neurobiobank.nih.gov/
