# Supplementary material for: Uric Acid as a Potential Peripheral Biomarker for Disease Features in Huntington’s Patients
Source: Front Neurosci. 2020 Mar 4;14:73. doi: 10.3389/fnins.2020.00073 (PMC7065265; doi:10.3389/fnins.2020.00073)
Supplement: Supplementary file 1 [file Data_Sheet_1.pdf]

Supplementary Material

Table S1. Summary of the clinical data for the subjects providing plasma samples

		CAG #	Age of Onset (yrs)	DBS	TFC	MMSE	TMS
**HD**	Mean	42.70	51.50	400.50	9.10	23.70	37.00
**N=38**	S.D.	2.87	12.40	109.10	2.69	2.87	17.24
	Min	37.00	33.00	102.00	3.00	20.00	5.00
	Max	49.00	80.00	712.50	13.00	30.00	70.00
**PM**	Mean	41.30	NA	240.50	12.87	27.93	2.19
**N=31**	S.D.	2.01	NA	72.70	0.56	1.74	2.13
	Min	38.00	NA	112.50	10.00	22.00	0.00
	Max	44.00	NA	375.00	13.00	30.00	7.00

Table S1. Continued - Summary of the clinical data for the subjects providing saliva samples.

		CAG #	Age of Onset (yrs)	DBS	TFC	MMSE	TMS
**HD**	Mean	42.67	52.15	403.10	8.87	23.85	37.67
**N=45**	S.D.	2.82	12.20	109.20	2.78	2.82	17.33
	Min	37.00	28.00	102.00	3.00	20.00	5.00
	Max	49.00	80.00	712.50	13.00	30.00	70.00
**PM**	Mean	41.00	NA	239.50	12.87	27.93	2.26
**N=49**	S.D.	1.87	NA	74.79	0.57	1.78	2.13
	Min	38.00	NA	112.50	10.00	22.00	0.00
	Max	44.00	NA	375.00	13.00	30.00	7.00

HD, Huntington's disease; PM, pre-manifest; SD, standard deviation. DBS, disease burden score; TFC, total functional capacity score. MMSE, mini-mental state exam; TMS, Total motor symptom score.

Table S2. Sex, age, and PMI effects on uric acid levels in post-mortem human prefrontal cortex.

	Sex		Age			PMI	
	p-value		r-value	p-value		r-value	p-value
Controls	0.078		-0.564	0.089		0.325	0.432
HD	0.435		0.333	0.374		-0.238	0.506
